# Modifications in FLAP's second cytosolic loop influence 5‐LOX interaction, inhibitor binding, and leukotriene formation

**DOI:** 10.1002/1873-3468.70066

**Published:** 2025-05-08

**Authors:** Erik Romp, Katharina Rataj, Stefanie König, Marcia E. Newcomer, Oliver Werz, Ulrike Garscha

**Affiliations:** ^1^ Department of Pharmaceutical/Medicinal Chemistry, Institute of Pharmacy Friedrich Schiller University Jena Germany; ^2^ Department of Pharmaceutical/Medicinal Chemistry, Institute of Pharmacy Greifswald University Germany; ^3^ Department of Biological Sciences Louisiana State University Baton Rouge LA USA

**Keywords:** complex formation, inflammation, leukotriene, lipid mediator, lipoxygenase, mutagenesis

## Abstract

Leukotrienes, synthesized via the 5‐lipoxygenase (5‐LOX) pathway in the arachidonic acid cascade, are critical in inflammation. Effective leukotriene production requires interaction between 5‐LOX and 5‐LOX‐activating protein (FLAP) at the nuclear membrane. This study used site‐directed mutagenesis to explore amino acid residues in FLAP's inhibitor binding pocket and cytosolic loops, assessing their impact on 5‐LOX product formation, the FLAP inhibitor MK886's efficacy, 5‐LOX translocation, and 5‐LOX/FLAP complex formation. Mutations in the second cytosolic loop, especially at residue S108, reduced MK886 potency and disrupted 5‐LOX/FLAP complex formation. These results highlight the second cytosolic loop of FLAP in the 5‐LOX/FLAP interaction and proper leukotriene formation and suggest that targeting this region could aid in the development of new FLAP inhibitors with improved pharmacokinetics.

## Graphical abstract

The enzyme 5‐lipoxygenase (5‐LOX) catalyzes the first step in the biosynthesis of leukotrienes (LTs) involved in inflammatory pathophysiology. After cellular stimulation, 5‐LOX translocates to the nucleus, interacting with the 5‐LOX‐activating protein (FLAP) to form LTA_4_ from arachidonic acid (AA). Here, we found that the C2‐loop, and in particular position S108 of FLAP, is crucial for the interaction with 5‐LOX.

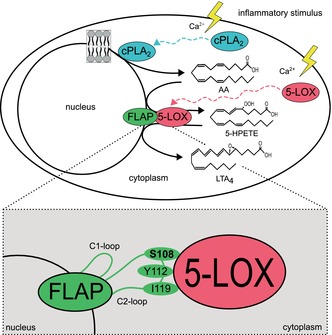

## Abbreviations

**5‐HPETE**, 5‐hydroperoxyeicosatetraenoic acid

**5‐LOX**, 5‐lipoxygenase

**AA**, arachidonic acid

**FLAP**, 5‐LOX‐activating protein

**HEK**, human embryonic kidney

**LT**, leukotrienes

**LTA**_**4**_, leukotriene A4

**PLA**, proximity ligation assay

**Fig. 1:**
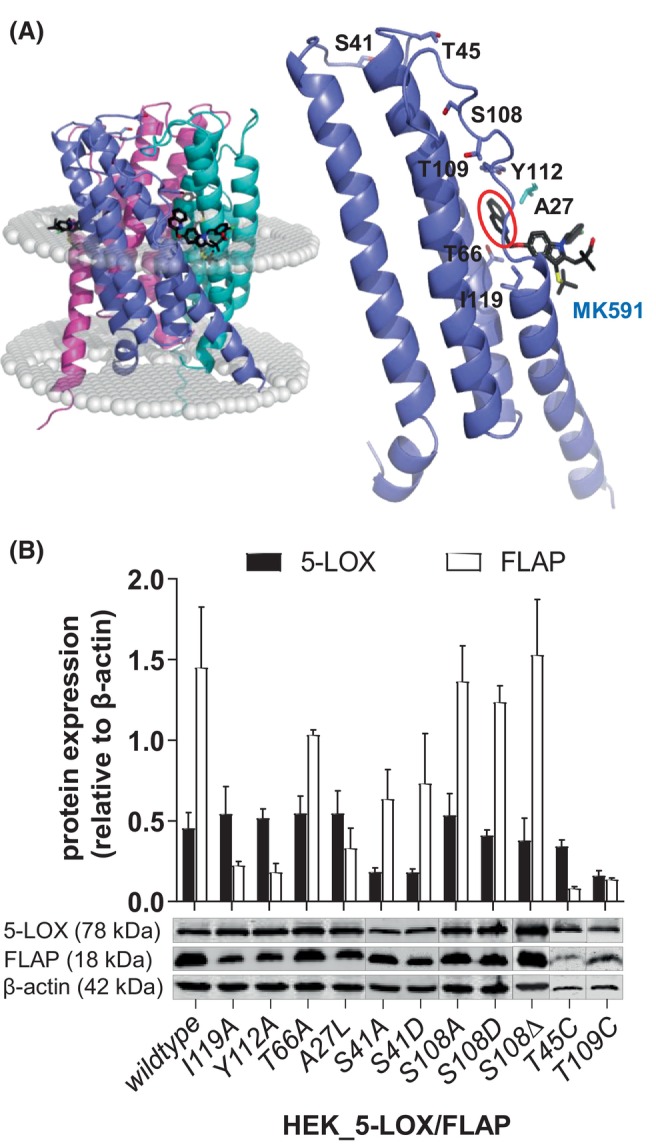
Generation of FLAP mutants and their expression in HEK293 cells with 5‐LOX. (A) (left) Cartoon rendering of the FLAP trimer with the limits of the hydrophobic region of the bilayer (according to Orientations of Proteins in Membranes) denoted with white spheres. MK591 is shown as stick rendering (black, C; red, O; blue, N; yellow, S; green, Cl). (right) The mutations made are depicted on a monomer and rotated to optimize the clarity of the labelling. A27 is contributed to the MK591 binding site by the neighboring monomer. The bulky aromatic substituent unique to MK591 is circled in red. (B) Western blot and densitometric analysis relative to β‐actin were used to assess 5‐LOX and FLAP co‐expression in stably transfected HEK293 cells. All data are given as mean ± SEM, *n* = 3.

Leukotrienes (LTs) are potent pro‐inflammatory lipid mediators involved in the pathophysiology of several inflammatory disorders, including asthma, cardiovascular diseases, cancer, and allergic conditions [1, 2, 3]. The initial step in LT formation is the conversion of arachidonic acid (AA) by the 5‐lipoxygenase (5‐LOX) to LTA_4_, which is the precursor for the downstream biosynthesis of LTB_4_ and cysteinyl LTs (cysLTs) [4]. The structure of 5‐LOX, consisting of 673 amino acids, was solved by crystallization of the enzyme stabilized by various modifications and discloses a catalytic domain containing the non‐heme iron and an amino‐terminal C2‐like domain, which is responsible for the Ca^2+^‐dependent membrane binding at the nuclear membrane [5, 6]. In intact cells, translocation of the cytosolic 5‐LOX to the nuclear membrane upon cellular stimulation and subsequent interaction with the nuclear membrane‐embedded 5‐lipoxygenase‐activating protein (FLAP) is essential for cellular LT biosynthesis [7, 8]. In this 5‐LOX/FLAP complex, FLAP functions as a binding and transfer protein for AA, which is released at the nuclear membrane by the cytosolic phospholipase A_2_ (cPLA_2_) [7, 9, 10]. Upon AA release and transfer to 5‐LOX, AA is dioxygenated in a two‐step mechanism to form 5‐hydroperoxyeicosatetraenoic acid (5‐HPETE), which is then converted to the unstable intermediate LTA_4_. Previous studies have shown that FLAP preferentially promotes the second step of catalysis, namely the formation of LTA_4_ from 5‐HPETE [11]. As a member of the membrane‐associated proteins in eicosanoid and glutathione metabolism (MAPEG) family [12], FLAP is a trimeric nuclear membrane protein, each monomer consisting of four transmembrane helices linked by two cytosolic loops and one luminal loop (Fig. 1A) [13]. Antagonists of the protein such as MK886 and MK591 (quiflapon) intercalate between the monomers within the nuclear membrane, compete for substrate binding, and disrupt the 5‐LOX/FLAP complex formation [10, 13] suggesting AA and 5‐HPETE as linkers for the 5‐LOX/FLAP interaction. MK886 is smaller than MK591; as in place of a quinoline‐2‐methoxy‐substituent, it has an isopropyl group. In the crystal structure of FLAP, the quinoline penetrates the helical bundle flanked by T66 and A27. Unfortunately, the complex of 5‐LOX and FLAP has not yet been crystallized, so the concrete site of interaction remains elusive. It is only suggested that the two proteins form a complex and physically interact, most likely via the cytosolic loops of FLAP, but this remains to be proven [14]. However, knowledge of the concrete regions and amino acids mediating the 5‐LOX/FLAP complex may help to design novel compounds that modulate LT biosynthesis to treat LT‐associated diseases. Here, we have carried out a site‐directed mutagenesis study of FLAP to decipher the residues that are critical for the interaction with 5‐LOX. Serine108 of the second cytosolic loop is revealed to be critical for 5‐LOX/FLAP interaction, as its mutation or deletion resulted in disruption of the complex and consequently, in reduced LT formation.

## Materials and methods

### Materials

Fetal calf serum (FCS), soybean trypsin inhibitor (STI) and the Duolink Proximity Ligation Assay (PLA) kit were from Merck (Darmstadt, Germany). DMEM, Penicillin/Streptomycin solution, and trypsin/EDTA were from GE Healthcare Life Science (Freiburg, Germany) and Sigma‐Aldrich (Taufkirchen, Germany). Geneticin G418 was from Roth GmbH (Karlsruhe, Germany) and GE Healthcare Life Science. Hygromycin B was from Roth GmbH or Invitrogen (Darmstadt, Germany). Leupeptin and phenylmethylsulfonyl fluoride (PMSF) were from Roth GmbH or Sigma‐Aldrich. Arachidonic acid (AA), calcium ionophore A23187, MK886, and prostaglandin B_1_ were from Cayman Chemical (Biomol, Hamburg, Germany). Zileuton was from Biozol (Eching, Germany) and Sequoia Research Products (Oxford, UK). The plasmid kits were from Qiagen (Hilden, Germany) and Thermo Fisher Scientific (Schwerte, Germany). Formaldehyde was from Thermo Fisher Scientific. The Lipofectamine LTX Plus Reagent and P3000 reagents, and One Shot TOP10 Chemically Competent *Escherichia coli* were also from Invitrogen. Ethanol was from Roth GmbH and Thermo Fisher Scientific. Glucose, bovine serum albumin (BSA) and calcium chloride were from Roth GmbH and AppliChem (Darmstadt, Germany). Glycerol was from Roth GmbH and Caesar & Loretz GmbH (Hilden, Germany). PBS was from Serva (Heidelberg, Germany). Acrylamide (30%) was from AppliChem and VWR (Darmstadt, Germany). TEMED was also from AppliChem. DMSO and bromophenol blue were from Roth GmbH and Merck (Darmstadt, Germany). Trifluoroacetic acid was from Merck and Sigma‐Aldrich. The Q5 Site‐Directed Mutagenesis kit was from New England Biolabs (Ipswich, MA, USA). The rabbit‐anti‐FLAP antibody was from Abcam (Cambridge, UK). The mouse‐anti‐5‐LOX antibody was from BD Biosciences (Heidelberg, Germany) and Proteintech Germany GmbH (Planegg‐Martinsried, Germany) and the rabbit‐anti‐β‐actin antibody was from Cell Signaling Technology Inc. (Danvers, MA, USA). Non‐immune goat serum, mounting medium/ProLong Diamond Antifade Mountant, and Alexa Fluor 488 goat anti‐rabbit, Alexa Fluor 555 goat anti‐mouse, and Alexa Fluor 647 goat anti‐mouse were from Abcam and Invitrogen. IRDye^®^ 680 LT goat‐anti‐rabbit and IRDye^®^ 800 CW goat‐anti‐mouse were from Li‐Cor Biosciences GmbH (Bad Homburg, Germany). The primers were designed by NEBaseChanger and purchased from TIB MIOLBIOL (Berlin, Germany). HPLC solvents were from VWR. All other chemicals were from Roth GmbH.

### Cell cultivation

HEK293 cells (ATCC: CRL‐1573, RRID: CVCL_0045) were cultured at 37 °C and 5% CO_2_ in DMEM supplemented with 10% heat‐inactivated FCS, 100 U·mL^−1^ penicillin, and 100 μg·mL^−1^ streptomycin. HEK293 cell lines stably expressing human 5‐LOX and/or human FLAP *wildtype* or mutants were selected using 400 μg·mL^−1^ geneticin G418 and/or 200 μg·mL^−1^ hygromycin B. All cell lines used in this study have been authenticated within the past 3 years. All experiments were performed with mycoplasma‐free cells.

### Site‐directed mutagenesis

Mutations in the FLAP coding sequence were introduced using the pcDNA3.1_FLAP plasmid as template, appropriate primers, and the Q5 Site‐Directed Mutagenesis kit (New England Biolabs) according to the manufacturer's instructions. Mutated plasmids were amplified in One Shot™ TOP10 Chemically Competent *E. coli* (Invitrogen) and plasmids were extracted using the GeneJET™ Plasmid Midiprep Kit (Fisher Scientific) or QIAGEN Plasmid Maxi Kit. All constructs were verified by sequencing.

### Stable expression of 5‐LOX and FLAP in HEK293

Transfection of HEK293 cells with pcDNA3.1 plasmids encoding for human 5‐LOX and human FLAP was performed using Lipofectamine™ LTX PLUS Reagent or Lipofectamine™ 3000 Reagent according to the manufacturer's instructions. HEK293 cells were grown to ~ 90% confluency in a 6‐well plate before the transfection mixture of pcDNA3.1_5LOX, lipofectamine, reagent, and Opti‐MEM was carefully added to the cells. After 24 h, the medium was replaced with fresh medium without antibiotics, and the cells were cultured for another 24 h. Stably transfected cells with pcDNA3.1_5LOX (G418) were selected by usage of 400 μg·mL^−1^ geneticin as described previously and co‐transfected with pcDNA3.1_FLAP (hygro) (*wildtype* and mutants). Co‐transfected cells (5‐LOX and FLAP) were selected by the addition of 200 μg·mL^−1^ hygromycin B. Protein expression of 5‐LOX and FLAP *wildtype* or mutants was verified by immunoblotting.

### SDS/PAGE and western blot analysis

Co‐transfected HEK293 cells were harvested by trypsinization and centrifugation. Pelleted cells were lysed with 100 μL Triton Lysis Buffer/1 × 10^7^ cells (20 mm Tris/HCl pH 7,4, 150 mm NaCl, 2 mm EDTA, 1% Triton‐X 100, 1 mm PMSF, 60 μg·mL^−1^ STI, 10 μg·mL^−1^ leupeptin) for 15 min on ice. Cell lysates were centrifuged and the supernatant was used for total protein quantification. Ten microgram of the samples were separated on 10% polyacrylamide gels (for 5‐LOX) and 16% gels (for FLAP). Proteins were transferred by wet‐tank blotting onto nitrocellulose membranes prior to incubation with primary antibodies (mouse anti‐5‐LOX, 1 : 1000; rabbit anti‐FLAP, 1 : 1000; mouse anti‐β‐actin, 1 : 1000) overnight at 4 °C. Secondary anti‐rabbit and anti‐mouse antibodies (IRDye^®^ 800 CW and IRDye^®^ 680 LT, 1 : 15 000) were applied for 1 h at room temperature (RT). Imaging of the immunoreactive bands was achieved by an Odyssey infrared imager (Li‐Cor Biosciences).

### Determination of 5‐LOX product formation in transfected HEK293 cells

To analyze cellular 5‐LOX product formation, HEK293 cells expressing 5‐LOX and FLAP *wild‐type* or mutants were harvested by trypsinization and pelleted by centrifugation (300 ***g***, 10 min, 4 °C). Cells were resuspended at a density of 1 × 10^6^ cells·mL^−1^ with PGC buffer (PBS/0.1% glucose, 1 mm CaCl_2_) and pre‐incubated with 300 nm MK886 or 0.1% DMSO (vehicle control, V/V) for 10 min at 37 °C. Two micromolar AA was added and the cells were stimulated with 2.5 μm Ca^2+^ ionophore A23187 for another 10 min at 37 °C. The reaction was terminated by the addition of 1 mL ice‐cold methanol until PGB_1_ (internal standard) and acidified PBS were added. Solid‐phase extraction (SPE) was performed before the analysis of 5‐LOX products by RP‐HPLC using a C‐18 Radial‐Pak column (Waters, Milford, MA, USA) with MeOH/water/trifluoroacetic acid as mobile phase [15].

### Subcellular localization of 5‐LOX and FLAP by immunofluorescence (IF) microscopy

Co‐transfected HEK293 cells were seeded onto poly‐d‐lysine‐coated coverslips (Kleinfeld Labortechnik GmbH; Neuvitro Corporation) at a cell density of 0.35 × 10^6^ cells/sample. Cells were cultured for 48 h at 37 °C and 5% CO_2_ until pre‐incubation with 300 nm MK886 or 0.1% DMSO for 10 min at 37 °C. After stimulation with 2.5 μm A23187 for 10 min at 37 °C, cells were fixed with 4% paraformaldehyde solution for 15 min. Cells were permeabilized with 100% acetone (5 min, 4 °C) and 0.25% Triton‐X (10 min, room temperature (RT)). Samples were incubated with primary antibodies against 5‐LOX (mouse anti‐5‐LOX, 1 : 100, 6A12 kindly provided by Prof. Dr. Steinhilber (Goethe University Frankfurt, Germany)) or from BD Biosciences and against FLAP (rabbit anti‐FLAP, 1 : 300; Abcam), each diluted in 10% non‐immune goat serum, overnight at 4 °C. After washing with PBS, fluorophore‐labeled secondary antibodies Alexa Fluor 488 goat anti‐rabbit (1 : 1000) and Alexa Fluor 555/647 goat anti‐mouse (1 : 1000/1 : 500) were applied for 1 h at RT in the dark. Samples were washed with PBS prior to staining of DNA in nuclei with DAPI and fixing on glass slides with mounting medium containing DAPI. Analysis of the subcellular localization of 5‐LOX and FLAP in HEK293 cells was achieved using a Zeiss Axiovert 200M or Axio Observer.Z1 microscope with a Plan‐APOCHROMAT 40×/1.3 Oil DIC (UV) VIS‐IR 0.17/∞ or Plan Neofluar 63×/1.25 Oil Ph3 DICII objective and an AxioCam MR camera (Carl Zeiss, Jena, Germany) for image acquisition.

### *In situ* analysis of 5‐LOX/FLAP complex formation by proximity ligation assay (PLA)

To analyze the *in situ* interaction of 5‐LOX and FLAP, a PLA assay (Duolink® PLA Technology, Sigma Aldrich) was performed according to the manufacturer's protocol. Cells were seeded, permeabilized, and fixed as described for IF microscopy. Samples were incubated with 10% non‐immune goat serum for 1 h at RT before incubation with primary antibodies overnight at 4 °C as mentioned. Specific secondary antibodies conjugated to oligonucleotides (PLA probe anti‐mouse MINUS and anti‐rabbit PLUS) were applied for 1 h at 37 °C. Ligase was added for 30 min at 37 °C to link oligonucleotides less than 40 nm in distance. The resulting DNA circle was amplified by rolling‐circle amplification by incubation with polymerase and fluorescently labeled oligonucleotides for 100 min at 37 °C. PLA images were taken after fixation of samples with DAPI‐containing mounting medium on glass slides, as described for IF microscopy. For quantitative evaluation of PLA images, the areas of the fluorescent signals were assessed. The DAPI‐stained nuclei emitted a blue signal, for which the area was determined. Within this area, the 5‐LOX/FLAP interactions were visible as magenta signals, and the corresponding area was quantified. The ratio of the two areas (magenta/blue) was calculated. Results were averaged over 50 images for each independent experiment (*n* = 4).

### Statistical analysis

Data are expressed as mean ± SEM of *n* independent experiments. Statistical analysis was performed using graphpad prism (GraphPad Software, Boston, MA, USA) with student's *t*‐test for evaluation. A *P* value of < 0.05 was considered statistically significant, indicated with *.

## Results

### Mutagenesis of putative FLAP residues involved in the interaction with 5‐LOX

The crystal structure of the FLAP trimer in complex with MK591 shows that each monomer consists of four transmembrane helices connected by two cytosolic and one luminal loop [13]. Most of the protein, including its inhibitor binding sites, is embedded in the nuclear membrane [13]. To identify the binding sites at FLAP for 5‐LOX, critical amino acid residues were mutated (Fig. 1A). These are amino acid residues located close to the inhibitor binding site of MK591, as well as serine and threonine residues within the cytosolic loop, which could serve as potential phosphorylation sites. Thus, residues within the membrane‐embedded helices like Ile119 and Thr66 have been replaced by alanine, transforming the bulky amino acid chains into small lipophilic units (*I119A*, *T66A*). The lipophilic Ala27 in proximity to the MK591 binding site was mutated to leucine (*A27L*) to create a steric hindrance at this position, and Tyr112, which is located at the transition from the C2‐loop to the α4‐helix, was changed to alanine (*Y112A*). Additionally, using the *NETPhos3.1* phosphorylation prediction software (https://services.healthtech.dtu.dk/services/NetPhos‐3.1/), Ser41 and Ser108 located within the cytosolic loop region were identified as putative phosphorylation sites with corresponding phosphorylation probability scores of 0.877 and 0.736, respectively. These two residues were mutated to aspartate to mimic the phosphorylation status or to alanine to change the structure more profoundly (*S41D*, *S108D*, *S41A*, *S108A*). Additionally, in one FLAP mutant, Ser108 was completely deleted (*S108Δ*). The residues Thr45 and Thr109, both located in the cytosolic loops in proximity to Ser41 and Ser108 and being potential targets for protein kinases, were mutated to cysteine (*T45C* and *T109C*) to maintain the structural integrity while inhibiting potential phosphorylation.

### 5‐LOX product formation in HEK293 cells stably expressing 5‐LOX and FLAP mutants

HEK293 cells were stably transfected with cDNA of 5‐LOX and FLAP *wildtype* or mutants, and selection of co‐transfected cells was achieved using geneticin and hygromycin B. Expression of 5‐LOX and FLAP at the protein level was verified by immunoblotting, with normalization to β‐actin (Fig. 1B). Compared to 5‐LOX, FLAP showed more inconsistent expression levels. This could also be attributed to site‐directed mutagenesis, which may have influenced the protein's recognition by the FLAP antibody or protein stability.

**Fig. 2:**
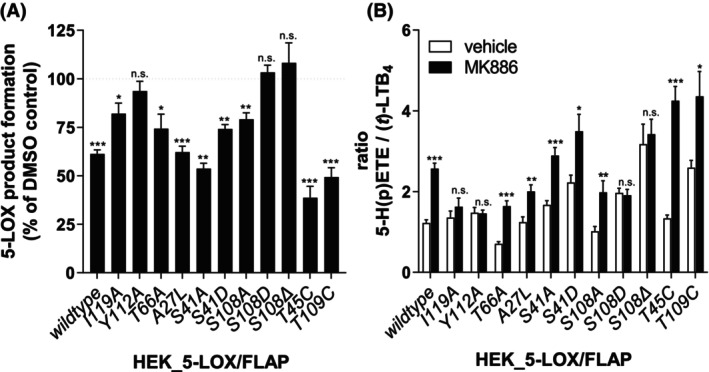
Impact of MK886 on 5‐LOX product formation in HEK293 cells co‐expressing 5‐LOX and FLAP *wildtype* or mutants. (A) Inhibition by MK886 presented as 5‐LOX product formation in % of the corresponding vehicle control (0.1% DMSO). (B) Ratio of formed 5‐H(P)ETE to formed *trans*‐isomers of LTB_4_ in the presence of MK886 or vehicle control of HEK293 cells stimulated with 2.5 μm A23187 plus 2 μm AA. All data are given as mean ± SEM, *n* = 5–7. Statistical analysis was performed using paired, two‐tailed Student's *t*‐test (n.s—not significant, ***P < 0.001, **P < 0.01, *P < 0.05 MK886 vs. the corresponding vehicle control).

The generated HEK293 cell lines expressing 5‐LOX and FLAP were assayed for 5‐LOX product formation in the presence of the FLAP inhibitor MK886 after stimulation with Ca^2+^ ionophore A23187 and low levels of exogenous AA (2 μm). MK886 is somewhat smaller than MK591, as in place of the quinoline‐2‐methoxy group it has an isopropyl group. In the crystal structure of FLAP, the quinoline penetrates the helical bundle flanked by T66 and A27. The total cellular formation of 5‐LOX products, including the intermediates 5‐H(P)ETE (alcohol and peroxide) and *trans*‐isomers of LTB_4_, appeared to be comparable to FLAP *wild‐type* cells and independent of most of the FLAP mutations (Table 1). Reduced amounts were detected only for the cell lines HEK_5‐LOX/FLAP(*S41D*) and HEK_5‐LOX/FLAP(*T109C*), which are presumably not associated with lower protein expression, as HEK_5‐LOX/FLAP(*S41A*) showed comparable expression levels to *S41D* but generated 5‐LOX product formation as wild‐type. However, the response to the FLAP inhibitor MK886 is of particular interest. Thus, LT biosynthesis in HEK_5‐LOX cells expressing FLAP *wild‐type* was reduced by 40% of the vehicle control, and most of the cell lines expressing the FLAP mutants were also susceptible to inhibition by the FLAP inhibitor, albeit to varying degrees (Fig. 2A, Table 1). The MK886‐suppressive effects against the FLAP mutants *A27L* and *S41A* were comparable to those against FLAP *wild‐type*, suggesting that the MK886 binding site remains intact on these variants. While S41 is on a surface loop, A27 lines the subcavity unique to the MK591. A lower but still significant 20–30% inhibition was observed for I119A, T66A, S41D, and S108A; MK886 inhibited *T45C* and *T109C* to 50–60% of the vehicle control.

**Table 1:** Levels of 5‐LOX products (ng per 10^6^ cells) in HEK293 cells, co‐expressing wildtype 5‐LOX and wildtype or mutants of FLAP after stimulation with 2.5 μm A23187 and 2 μm AA with 300 nm MK886 or vehicle control (0.1% DMSO), as indicated. All data are given as mean ± SEM, *n* = 5–7.

	5‐LOX product formation (ng per 10^6^ cells)	
	Vehicle	MK886
*Wild‐type*	88.9 ± 5.0	54.6 ± 4.0
*I119A*	79.0 ± 7.9	64.3 ± 6.2
*Y112A*	80.2 ± 13.7	73.3 ± 9.5
*T66A*	92.5 ± 6.5	67.7 ± 6.6
*A27L*	77.4 ± 4.5	48.3 ± 4.3
*S41A*	64.0 ± 7.4	34.4 ± 4.4
*S41D*	31.6 ± 5.0	23.2 ± 3.5
*S108A*	87.3 ± 6.0	69.0 ± 4.9
*S108D*	75.3 ± 11.4	78.2 ± 12.8
*S108Δ*	64.5 ± 3.4	68.6 ± 4.0
*T45C*	60.3 ± 1.9	23.6 ± 4.1
*T109C*	41.3 ± 6.4	22.0 ± 5.3

Strikingly, MK886 did not affect 5‐LOX product formation in HEK_5‐LOX/FLAP(*Y112A*), HEK_5‐LOX/FLAP(*S108D*) and HEK_5‐LOX/FLAP(*S108Δ*) cell lines. Notably, mutants with reduced (*I119A*; 18.6% inhibition) or abolished (*Y112A*, *S108D*, *S108 Δ*) inhibitory capacity are located in the second cytosolic loop, suggesting a critical region for the action of MK886. FLAP promotes the second step in LT biosynthesis to convert 5‐H(P)ETE to LTA_4_; thus, the ratio of 5‐H(P)ETE to LTB_4_‐isomers reflects the influence of FLAP on LT biosynthesis. FLAP inhibition results in a higher production of the hydroperoxide intermediate, increasing the ratio in favor of 5‐H(P)ETE formation [11] (Fig. 2B). Consistent with prior data for the protein expression in HEK cells, the wildtype cell line shows a 5‐H(P)ETE/*trans*‐LTB_4_ ratio of about 1.2. Co‐expression of 5‐LOX with FLAP mutants *I119A* (1.4), *Y112A* (1.5), *A27L* (1.2), *S41A* (1.7), *S108A* (1.0), and *T45C* (1.3) resulted in comparable intermediate/product ratios. However, the HEK293_5‐LOX/FLAP(*T66A*) cells exhibit a decreased ratio of 0.7, implying pronounced LTA_4_ formation. In contrast, the cell lines with FLAP mutants *S41D* (2.3), *S108D* (2.0), *S108 Δ* (3.2) and *T109C* (2.6) showed elevated ratios compared to FLAP *wildtype*, reflecting diminished LTA_4_ formation and consistent with an impaired 5‐LOX/FLAP interaction (Fig. 2B). Pretreatment with MK886 resulted in an increased 5‐H(P)ETE/*trans*‐LTB_4_ ratio in the HEK293 cell lines expressing 5‐LOX together with FLAP *wildtype*, *T66A*, *A27L*, *S41A*, *S41D*, *S108A*, *T45C*, or *T109C*, indicating that inhibitor sensitivity has not been impaired. In contrast, for mutations located in the C2‐loop of FLAP (*I119A*, *Y112A*, *S108D*, *S108Δ*) (except for the *S108A* mutant) the presence of MK886 did not change the intermediate/product ratio. The loss of sensitivity to MK886 with mutations outside the drug‐binding site would be expected if these mutations impair the 5‐LOX/FLAP interaction. In summary, alterations in the C2‐loop of FLAP, especially of the S108 residue, are critical for LTA_4_ formation and proper inhibitor binding.

### FLAP mutagenesis does not affect the subcellular distribution of 5‐LOX

**Fig. 3:**
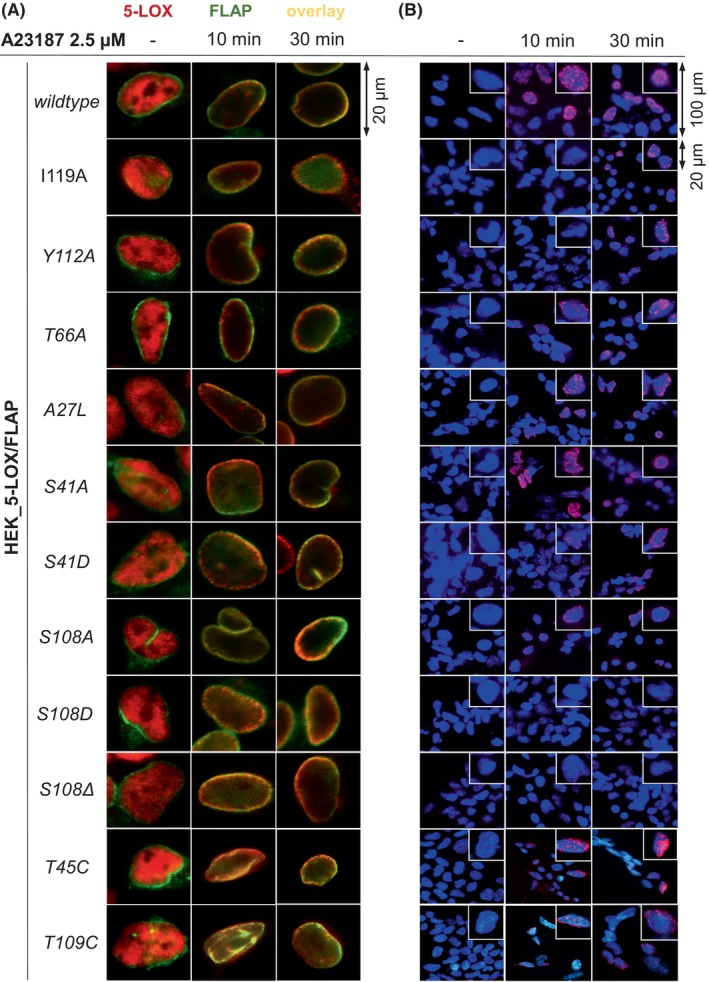
Mutagenesis of FLAP affects 5‐LOX/FLAP complex formation at the nuclear envelope, but not 5‐LOX translocation. HEK293 cells stably co‐expressing 5‐LOX and FLAP (wildtype or mutants) were stimulated with 2.5 μm A23187 at 37 °C for 10 or 30 min. (A) Subcellular localization of 5‐LOX (Alexa Fluor 555 or 647, red) was determined by IF microscopy. FLAP wildtype or mutants were analyzed with Alexa Fluor 488, green. Images are representative for three independent experiments. (B) *In situ* 5‐LOX/FLAP‐interaction was determined by proximity ligation assay (PLA). Protein–protein interactions are shown as magenta spots within an overlay image with DAPI‐stained nuclei (blue). Overview images (100 μm) and selected cells (20 μm) are representative of at least three independent experiments.

The translocation of 5‐LOX from the cytosol or nucleosol to the nuclear membrane is a prerequisite for forming the 5‐LOX/FLAP complex. Here, we investigated whether FLAP mutants interfere with the subcellular redistribution of 5‐LOX in A23187‐activated HEK293 cells by immunofluorescence microscopy (Fig. 3A). 5‐LOX translocated to the nuclear membrane in all HEK293 cell lines studied, regardless of the mutations within FLAP. The co‐localization of 5‐LOX and FLAP was observed after 10 min of stimulation with A23187 and was not altered after 30 min.

### FLAP mutants within the second cytosolic loop hamper 5‐LOX/FLAP interaction *in situ*

**Fig. 4:**
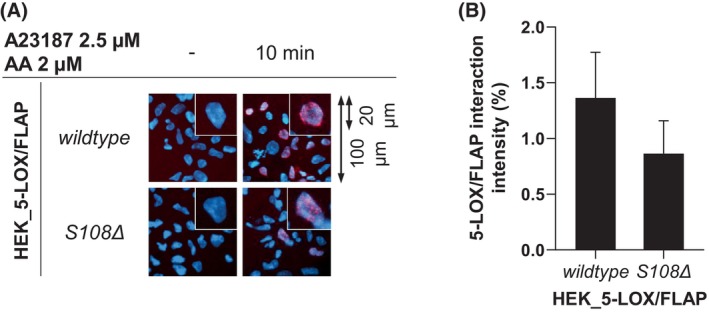
AA restores 5‐LOX/FLAP complex formation in HEK_5‐LOX/FLAP(S108∆). HEK293 cells stably co‐expressing 5‐LOX and FLAP (*wildtype* or S108∆) were stimulated with 2.5 μm A23187 and 2 μm AA at 37 °C for 10 min. *In situ* 5‐LOX/FLAP interaction was analyzed by PLA. (A) Protein–protein interactions are shown as magenta spots within an overlay image with DAPI‐stained nuclei (blue). Overview images (100 μm) and selected cells (20 μm) are representative of at least four independent experiments. (B) For each replicate, PLA was quantitatively evaluated in 50 images (mean ± SEM, *n* = 4). The area of cell nuclei (DAPI, blue signal) and the area of 5‐LOX/FLAP interactions (red signal) located within nuclei were determined. The areas of the interactions were then compared with the areas of the nuclei.

Next, the 5‐LOX/FLAP complex formation *in situ* was analyzed using the immunofluorescence‐based proximity ligation assay (PLA) (Fig. 3B). The assay enables the analysis of protein–protein interactions within a distance of less than 40 nm and has previously been validated for the 5‐LOX/FLAP complex, as described [10]. Unstimulated HEK293 cells expressing wildtype 5‐LOX and FLAP did not show an interaction signal, as expected. Stimulation of the cells with A23187 resulted in the complex formation of all FLAP mutants with 5‐LOX after 30 min, except for the *S108D* and *S108Δ* mutants. Fluorescence signals of the interaction of the FLAP mutants *T66A*, *A27L*, *S41A*, *S41D*, *S108A*, *T45C*, and *T109C* with 5‐LOX were comparable to those of FLAP *wildtype*. However, mutants *I119A* and *Y112A*, positioned in the C2‐loop and near *S108*, exhibit a delayed interaction, evidenced by weaker signals observed after 10 min of stimulation compared to the wildtype. However, after 30 min of stimulation, the interaction signal became comparable to cells with wildtype FLAP. It is noteworthy that this was not observed for *S108D* and *S108Δ* mutants, where prolonged stimulation did not lead to any interaction with 5‐LOX, implying prevention of the formation of the 5‐LOX/FLAP complex. Previous interaction studies in blood leukocytes suggest that AA and/or the intermediate 5‐HPETE facilitate complex formation [10]. However, stimulation of HEK293 cells with A23187 leads to only minimal AA release. Therefore, the *in situ* interaction of 5‐LOX with the FLAP *S108Δ* mutant was investigated in the presence of exogenous AA (2 μm). Interestingly, the disturbed interaction could be restored when 2 μm AA was added, confirming that AA as substrate is an important linker between the 5‐LOX/FLAP complex (Fig. 4).

## Discussion

The biosynthesis of LTs entails the involvement of a spectrum of enzymes and proteins distributed across cellular compartments [3]. In the initiation of LT biosynthesis, 5‐LOX undergoes translocation to the nuclear membrane to accomplish the formation of LTA_4_, the primary LT in the cascade. Although the exact mechanism by which FLAP binds and transports AA remains elusive, it has been proposed that AA diffuses through the membrane and enters FLAP via the bilayer. Subsequently, the complex assembly of 5‐LOX with FLAP facilitates the reorientation of AA from FLAP to the active site of 5‐LOX [7, 16, 17]. Consequently, mutations in FLAP that affect the binding of AA or the 5‐HPETE intermediate could disrupt these processes. The binding of AA and the 5‐LOX/FLAP interaction can be prevented by FLAP inhibitors [10], whose binding site overlaps with the AA binding pocket, indicating FLAP's potential as a pharmacological target [13]. However, no clinically applicable FLAP inhibitor has yet made it to the pharmaceutical market [18]. One possible reason could be the lipophilic nature of all inhibitors that need to compete with AA for the binding site, which comes with the drawback of unfavorable pharmacokinetic properties. Moreover, the crystal structure of the 5‐LOX/FLAP complex has yet to be elucidated, and the specific residues within FLAP that are crucial for forming the complex with 5‐LOX remain unresolved. Consequently, we sought to investigate whether targeted mutagenesis of FLAP would affect *in situ* interaction with 5‐LOX, cellular LT biosynthesis, and the influence of MK886's inhibitory capability.

FLAP is embedded in the nuclear membrane and the crystal structure revealed a binding mode for the lipophilic inhibitor MK591 and by extension its MK886 analogue between adjacent monomers of the FLAP trimer [13]. Here, we investigated residues in FLAP which form hydrophobic (A27, α1‐helix; Y112, C2‐loop, I119, α4‐helix) and polar (T66, α2‐helix) interactions with inhibitors to analyze whether inhibitor or substrate binding influences LT formation and 5‐LOX/FLAP complex formation. Previous radioligand binding assay revealed impaired binding of MK591 for the *A27V*, *T66A*, and *Y112A* mutants [13]. These three amino acids surround the quinoline ring that is unique to MK591, with A27 and T66 deep in a subcavity of the bulk inhibitor. In contrast, Y112 is at the membrane interface and its position in the co‐crystal structure may be driven by the presence of an inhibitor. In the present study, *A27L* and *T66A* mutants did not affect cellular LT biosynthesis, the inhibitory capacity of MK886, or the 5‐LOX/FLAP interaction. However, mutation of Y112 (*Y112A*) or I119 (*I119A*), which appear to demarcate the MK591 binding site result in the loss of MK886's inhibitory capacity. This is partly in line with previous radioligand binding studies that confirmed the importance of Y112 for inhibitor binding, but not of I119 [13]. In addition, 5‐LOX/FLAP complex formation detectable by PLA appeared to be prevented in these mutants, as the PLA signal could only be detected after 30 min of stimulation, at a time when LTA_4_ formation was already complete. Note, that the 5‐LOX product formation remained unaffected by these mutations. LTA_4_ is produced in HEK cells which express only 5‐LOX; therefore, the presence of non‐functional FLAP would not be expected to completely abrogate the generation of LT [11]. However, the crystal structure is consistent with the positioning of Y112 and I119 within the C2‐loop impacting both inhibitor binding and complex formation at the nuclear membrane. According to the Orientation of Proteins in Membrane server predictions [19], only I119 is buried in the hydrophobic portion of the bilayer, just below the aqueous phase.

FLAP is a member of the MAPEG family along with LTC_4_ synthase (LTC_4_S), microsomal PGE_2_ synthase (mPGES) and microsomal glutathione S‐transferases (MGST‐1/2/3). Although they have limited sequence homology, the secondary structure within the MAPEG family is largely conserved [20]. In 2016, Ahmad *et al*. [21] identified Ser36 as a phosphorylation site within a cytosolic loop of LTC_4_S. It has been proposed that phosphorylation pulls the loop towards an adjacent subunit, forming a hydrogen bond with Arg104 and leading to a reduction in catalytic activity of LTC_4_S [21]. Using a phosphorylation prediction tool for FLAP, S41, T45 of the C1 loop, and S108 and T109 of the C2 loop were suggested to be post‐translationally phosphorylated. Thus, we systematically mutated serine residues to phospho‐deficient (*S41A*, *S108A*) and phosphomimetic (*S41D*, *S108D*) residues, and we changed threonines to non‐phosphorylatable cysteines (*T45C*, *T108C*). The overall 5‐LOX product formation was comparable for all FLAP mutants and ranged between 36 and 92 ng/10^6^ cells. Note that variations in product formation may arise from differences in cell populations, where protein expression levels and exogenous AA supply may not be precisely equal across all samples. All phospho‐deficient mutants and the phosphomimetic mutant *S41D* showed comparable behavior to *wildtype* FLAP in terms of susceptibility towards MK886 and 5‐LOX/FLAP interaction. This let us conclude that potential post‐translational phosphorylation by cellular kinase is not mandatory for LT complex assembly and does not influence MK886 binding. Furthermore, the C1 loop does not seem to play a critical role in 5‐LOX/FLAP interaction, as all mutants in that region (*S41A*, *S41D*, *T45C*) did not impact the PLA signal.

## Concluding remarks

Upon close examination of the 5‐LOX products formed, it becomes evident that cells expressing the *S108Δ* and *T109C* mutants exhibit an increased ratio of 5‐H(P)ETE to LTB_4_ isomers. This suggests a reduction in LTA_4_ formation, indicative of compromised FLAP function. Such impairment in FLAP function hinders the second step of 5‐LOX product formation, namely LTA_4_ biosynthesis [11]. S108 plays an extraordinary role in FLAP recognition, as the phosphomimetic *S108D* and the deletion mutant *S108Δ* resulted not only in impaired LTA_4_ formation but additionally abolished the inhibitory effect of MK886, despite their positioning distal to the inhibitor binding site. Furthermore, these mutations led to an inhibition of the 5‐LOX/FLAP complex assembly in the experimental HEK‐cell system, which could be restored by the addition of exogenous AA. This observation confirms the hypothesis that the complex assembly is facilitated and orientated by AA or the intermediate, as already suggested by previous studies [10]. The significance of the C2‐loop region was hinted at before, as FLAP mutants lacking residues 106 to 108 led to a higher 5‐H(P)ETE / LTB_4_ ratio in SF9 cells [22]. Our present data unequivocally demonstrate that the C2‐loop, and particularly the S108, serves as the pivotal amino acid. Nevertheless, the question arises whether cellular phosphorylation at S108 may attenuate LT biosynthesis and complex assembly. Unfortunately, despite intense effort by a phosphoproteomic approach, phosphorylated FLAP could not be detected in our study. The reason could stem from factors such as insolubility, low abundance, rapid dephosphorylation, or the complex fragmentation and ionization behavior of membrane proteins [23]. Altogether, we identified the second cytosolic loop, and especially S108, as a crucial residue for 5‐LOX/FLAP complex assembly and efficient LT biosynthesis. With this study, we present S108 and the C2 loop as a novel region of FLAP that is amenable to pharmacological targeting to interfere with LT biosynthesis. This discovery may pave the way for identifying novel inhibitors with advantageous pharmacokinetic properties.

## Author contributions

ER contributed to conceptualization, methodology, software, validation, formal analysis, investigation, writing—review and Editing. KR contributed to conceptualization, methodology, software, validation, formal analysis, investigation, writing—review and editing. SK contributed to formal analysis, review and editing. MEN contributed to conceptualization, methodology, writing—review and editing, visualization. OW contributed to writing—review and editing. UG contributed to conceptualization, writing—review and editing, supervision, project administration, and funding acquisition.

## Peer review

The peer review history for this article is available at https://www.webofscience.com/api/gateway/wos/peer‐review/10.1002/1873‐3468.70066.

## Data Availability

All data supporting the findings of this study are available upon request from the corresponding author.
